# Human T-lymphotropic virus 1/2 infection among immigrants and refugees in Central Brazil, an emerging vulnerable population

**DOI:** 10.3389/fpubh.2023.1265100

**Published:** 2023-10-06

**Authors:** Thaís Augusto Marinho, Larissa Silva Magalhães, Kamila Cardoso dos Santos, Thaynara Lorrane Silva Martins, Grazielle Rosa da Costa e Silva, Ágabo Macedo da Costa e Silva, Megmar Aparecida dos Santos Carneiro, Karlla Antonieta Amorim Caetano, Sheila Araújo Teles, Regina Maria Bringel Martins

**Affiliations:** ^1^Institute of Tropical Medicine and Public Health, Federal University of Goiás, Goiânia, Brazil; ^2^Faculty of Nursing, Federal University of Goiás, Goiânia, Brazil

**Keywords:** HTLV, Venezuelans, Haitians, migration, Brazil

## Abstract

**Introduction:**

Migratory flows play a significant role in the spread of human T-lymphotropic virus 1/2 (HTLV-1/2). In the last decade, a substantial migration of individuals occurred from Haiti and Venezuela to Brazil. However, data on the prevalence of HTLV-1/2 infection among these international migrants in Brazil are scarce. This study describes the prevalence of this infection among immigrants and refugees in Central Brazil.

**Methods:**

A cross-sectional study was conducted with 537 international migrants in the State of Goiás, Central Brazil. Participants were interviewed, and blood samples were collected. Serological screening for anti-HTLV-1/2 was performed using an enzyme-linked immunosorbent assay (ELISA; Murex HTLV-I + II, DiaSorin, Dartford, UK), and seropositive samples were submitted for confirmation by a line immunoassay (INNO-LIA HTLV I/II, Fujirebio, Europe N.V., Belgium).

**Results:**

The majority of participants were males (54.4%), between 18 and 50 years old (78%; mean age: 29.1 years), self-declared black (55.1%), reported 1 to 12 years of formal education (70.9%), and were either Venezuelans (47.9%) or Haitians (39.7%). Additionally, 50.1% were immigrants, 49% were refugees, and five were Brazilian children (0.9%) born to Haitian immigrant parents. The overall prevalence of anti-HTLV-1/2 was 0.95% (95% CI: 0.31–2.28), with HTLV-1 at 0.19% and HTLV-2 at 0.76%. All seropositive individuals (*n* = 5) were refugees from Venezuela, resulting in a rate of 2.26% for anti-HTLV-1/2, HTLV-1 (0.45%) and HTLV-2 (1.81%) among Venezuelan refugees. Of the demographic and behavioral characteristics evaluated, unprotected sexual intercourse and having more than one sexual partner (≥2) in the previous 12 months were associated with HTLV-1/2 seropositivity among Venezuelans.

**Conclusion:**

This study revealed, despite the low seroprevalence of HTLV-1/2 among international migrants in Central Brazil, evidence of HTLV-1 and HTLV-2 infections in Venezuelan refugees. In addition, their characteristics highlight that specific social and health programs should be implemented for these emergent and socially vulnerable migrant groups.

## Introduction

The human T-lymphotropic virus type 1 (HTLV-1) is a retrovirus associated with severe diseases worldwide, including adult T-cell leukemia/lymphoma (ATL) and progressive tropical spastic paraparesis/HTLV-1-associated myelopathy (TSP/HAM). Additionally, other inflammatory diseases are associated with this virus (1), while HTLV-2 has been associated with rare cases of myelopathy (2).

Despite estimates that at least 5–10 million people worldwide are infected with HTLV-1, its epidemiology is poorly understood (1). Regarding the HTLV-2, it is estimated that 800,000 (range 670,000-890,000) people globally are infected with this virus, with a remarkably high prevalence in American indigenous populations and among people who inject drugs (3). These retroviruses are transmitted by unprotected sexual intercourse, transfusion/transplantation of contaminated blood/organs, or injecting drugs, and from mother to child, mainly by breastfeeding (1, 4).

Currently, it is estimated that 281 million people are international migrants worldwide (5), and approximately 108.4 million individuals were forced to flee their homes at the end of 2022, of whom 35.3 million were refugees. In Latin America, significant migration and asylum-seeking activities have been motivated by social, political, and economic crises, especially by Venezuelans in recent years (6). In Brazil, around 1.4 million people were international migrants in 2021, most of whom were from Latin American countries, especially Venezuela and Haiti (7).

Migratory flows contribute to the emergence of infectious agents in new geographic areas; and have indeed played a significant role in the introduction and spread of HTLV-1 and HTLV-2 in Brazil (8). High HTLV-1 prevalence rates were found among Japanese immigrants and descendants in Campo Grande, Mato Grosso do Sul (6.8%), and São Paulo (5.1%) (9, 10). In addition, a high prevalence of HTLV-1/2 infection (3.0%; HTLV-1: 1.0% and HTLV-2: 2.0%) was observed among Warao indigenous refugees from Venezuela living in Belém, Pará (11). However, there remains a paucity of data on HTLV-1/2 prevalence among the broader international migrant population in Brazil, which includes individuals of diverse geographical, social, and cultural origins. Therefore, this study aims to investigate the prevalence of HTLV-1/2 infection among immigrants and refugees in Central Brazil.

## Methods

### Study population

A cross-sectional study was conducted among international migrants (immigrants and refugees) residing in the State of Goiás, Central Brazil. Thus, the study was performed in three municipalities in Goiás, which had received most international migrants during the study, including Goiânia (the capital of the State of Goiás), Aparecida de Goiânia, and Anápolis, between July 2019 and May 2021 (due to the COVID-19 pandemic, collections were interrupted during local social distancing and lockdown compulsory measures).

For this study, the term international migrant is an umbrella term used to refer to any person living in a country other than where they were born (12), including both immigrants and refugees. An immigrant is understood to be any person who migrates to a country other than where they were born to establish residence for a variety of reasons. A refugee includes any person who left their country due to a well-founded fear of being persecuted or another situation that affects human rights and is forced to move to preserve his/her life or freedom under vulnerable conditions and needs international protection (13, 14).

During the data collection period, there were no data on HTLV-1/2 prevalence among international migrants from Latin America in Brazil for up to 10 years. Therefore, the sample size was estimated to be 681 individuals, considering a hypothetical prevalence of 1.5% for HTLV-1/2 infection, a significance level of 95% (*α* < 0.05), 80% statistical power (*β* = 20%), a precision of 1% and an effect design of 1.2%. However, a convenience sample of 537 individuals was obtained (80% of the target sample) due to the lack of knowledge of the exact number of immigrants and refugees in Goiás, the difficulty of accessing them, and linguistic and cultural challenges in addition to the COVID-19 pandemic. The inclusion criteria for this study were: being an international migrant in Goiás and having lived in Brazil for up to 10 years. In addition, children aged less than 2 years were excluded due to the difficulty of drawing their blood samples.

### Ethical aspects and sample collection

This study was approved by the Committee on Ethics in Research of the Federal University of Goiás (protocol number: 06871019.7.0000.5083). All subjects who voluntarily agreed to participate in the study signed an informed consent form.

Data and blood samples were collected at private locations, such as churches and non-governmental organization facilities. Data collection instruments were prepared in Portuguese, English, and the native languages of the participants in the study (Spanish, French, and Creole/Haiti). In addition, interviewers fluent in these languages were trained for this data collection by the project team. Participants were interviewed face-to-face by trained interviewers in their language after they agreed to participate in the study and signed the Free and Informed Consent Form. Those under 18 years of age were consented using the Terms of Informed Consent of Children and Adolescents and Free and Informed Assent.

A total of 537 international migrants agreed to participate in the study and were interviewed using a structured script containing questions about sociodemographic data, risk characteristics associated with HTLV infection, and also about their medical history. Then, a blood sample (10 ml) was collected from all participants.

### Laboratory tests

Of the 537 international migrants who consented to participate, a total of 528 (98.3%) serum samples were tested for anti-HTLV-1/2 (Murex HTLV-I + II, DiaSorin, Dartford, UK) using an enzyme-linked immunosorbent assay (ELISA). Reactive samples were confirmed using a line immunoassay (INNO-LIA HTLV I/II, Fujirebio, Europe N.V., Belgium). Samples that tested positive by LIA were considered positive for HTLV-1 or 2 infections.

### Data analysis

The collected data were analyzed using the Statistical Package for the Social Sciences (SPSS) version 20.0. Descriptive analyses were performed using frequency distributions, mean values, and standard deviations. Prevalence of anti-HTLV-1/2 was calculated using a 95% confidence interval (95% CI). Fisher’s exact test was used to evaluate the association of demographic and behavioral characteristics with the presence/absence of HTLV-1 or HTLV-2 among Venezuelan migrants who were tested for anti-HTLV-1/2 (*n* = 254). All Venezuelans in the study reported being breastfed during childhood, which precluded further statistical analysis on this variable. For this study, *p*-values <0.05 were considered statistically significant.

## Results

**Figure 1:**
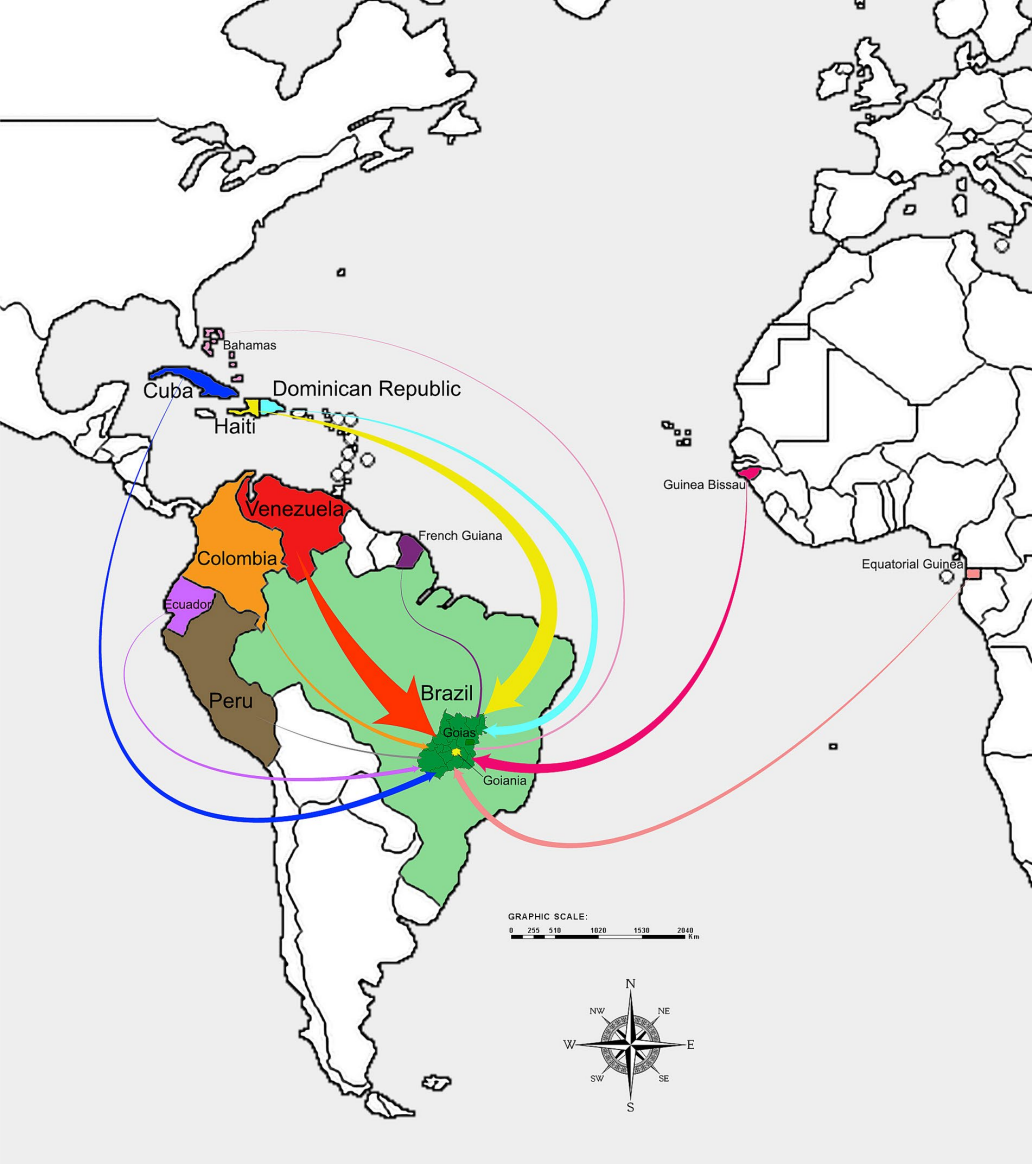
This map represents the migratory flow of people from their countries of origin to the State of Goiás (Central Brazil). The width of the arrows indicates the volume of migration from each of the 11 countries included in this study, with wider arrows representing a larger flow of migrants.

Table 1 presents the sociodemographic characteristics of the study population. The majority were male (54.4%), between 18 and 50 years old (78%; mean and standard deviation of 29.1 ± 12.5 years), self-declared black (55.1%), single (52.5%), and reported 1 to 12 years of formal education (70.9%; mean and standard deviation of 10.3 ± 4.7 years). Half of the participants were immigrants (50.1%), 49% were refugees, and five were Brazilian children (0.9%) born to Haitian immigrant parents. Regarding their professional situation, 38.2% reported working with permanent contracts, 14.4% with temporary contracts, self-employed, or occasional work, and 35.9% were unemployed. The majority of migrants were Venezuelans (47.8%) and Haitians (39.7%). The remaining (11.6%) comprised Bissau-Guineans (*n* = 24), Dominicans (*n* = 18), Ecuadorians (*n* = 7), Cubans (*n* = 5), Equatorial Guineans (*n* = 3), Colombians (*n* = 2), Peruvian (*n* = 1), French Guianese (*n* = 1), and Bahamian (n = 1) (Figure 1).

**Table 1:** Sociodemographic characteristics of the study participants.

**Variable**	***N* = 537**	**%**
**Sex**		
Male	292	54.4
Female	245	45.6
**Age (years)**		
<18	90	16.8
18–30	212	39.5
31–50	207	38.5
≥ 51	28	5.2
**Color**		
Black	296	55.1
Brown	145	27.0
White	72	13.4
Indigenous	24	4.5
**Marital status**		
Single	282	52.5
Married/living with partner	229	42.6
Separated/Widowed	15	2.8
Not reported	11	2.1
**Education (years)**		
<10	200	37.2
10–12	181	33.7
>12	142	26.5
Not reported	14	2.6
**Immigration status in Brazil**		
Immigrant	269	50.1
Refugee	263	49.0
Brazilian	5	0.9
**Professional situation in Brazil**		
Permanent contract	205	38.2
Temporary contract	37	6.9
Self-Employed	30	5.6
Occasional Work	10	1.9
Unemployed	193	35.9
Student	51	9.5
Child, not school aged	11	2.0
**Country of birth**		
Brazil (1st Generation Children)	5	0.9
Venezuela	257	47.8
Haiti	213	39.7
Guinea-Bissau	24	4.5
Dominican Republic	18	3.3
Ecuador	7	1.3
Cuba	5	0.9
Equatorial Guinea	3	0.6
Colombia	2	0.4
Peru	1	0.2
French Guiana	1	0.2
Bahamas	1	0.2

Among the 528 international migrants in Central Brazil tested for anti-HTLV-1/2 antibodies, five (0.95%; 95% CI: 0.31–2.28) were found to be anti-HTLV-1/2 positive by ELISA. After confirmatory testing (LIA), one was positive for HTLV-1 (0.19%), and four were positive for HTLV-2 (0.76%). All HTLV-1/2 seropositive individuals did not report any symptoms. They were all refugees from Venezuela, resulting in a rate of 2.26% (5/221) for anti-HTLV-1/2, HTLV-1 (0.45%) and HTLV-2 (1.81%) among Venezuelan refugees.

As shown in Table 2, the HTLV-1 seropositive individual was an 18-year-old male, an indigenous Venezuelan refugee who was breastfed during childhood (>6 months). He reported condom use with his sole sexual partner in the last 12 months. He denied having received a blood transfusion or injected drugs. The ages of the four HTLV-2 seropositive Venezuelan refugees ranged from 23 to 57 years; two were males, and two were females, one of whom was indigenous. They were breastfed during childhood (> 6 months) and reported not injecting drugs or receiving blood transfusions. Regarding their sexual characteristics, all reported not having used a condom with one or two sexual partners in the last 12 months. However, none reported sex for money or a previous diagnosis of any sexually transmitted infections (STI).

**Table 2:** Characteristics of HTLV-1/2-positive international migrants in Central Brazil.

**Characteristics**	**Individual**				
	**M-77**	**M-69**	**M-208**	**M-352**	**M-440**
Sex	M	F	M	F	M
Age (years)	18	23	36	53	57
Color	Indigenous	Indigenous	Brown	Brown	Brown
Marital status	Single	Single	Married	Single	Single
Education (years)	9	4	9	16	12
Income (n. of minimum wages)	0	1	1	<1	0
Country of origin	Venezuela	Venezuela	Venezuela	Venezuela	Venezuela
Migratory status	Refugee	Refugee	Refugee	Refugee	Refugee
Breastfed (>6 months)	Y	Y	Y	Y	Y
Blood transfusion	N	N	N	N	N
Injecting drug use	N	N	N	N	N
Sexually active	Y	Y	Y	Y	Y
Sex for Money	N	N	N	N	N
Use of condoms*	Y	N	N	N	N
Number of sexual partners*	1	1	1	2	2
History of STI	N	N	N	N	N
Type of HTLV	HTLV-1	HTLV-2	HTLV-2	HTLV-2	HTLV-2

F, female; M, male; N, no; Y, yes; STI, sexually transmitted infections; *in the last 12 months.

Table 3 displays the demographic and behavioral characteristics evaluated, where unprotected sexual intercourse and having more than one sexual partner (≥2) in the last 12 months were associated with HTLV-1/2 seropositivity among Venezuelans. Seropositivity was significantly lower among those who reported condom use during sexual intercourse in the last 12 months compared to those who did not (0.5% vs. 33.3%, *p* < 0.001). Additionally, seropositivity was higher among those who reported ≥2 sexual partners in the last 12 months than those with only one sexual partner (13.3% vs. 1.6%, *p* = 0.044).

**Table 3:** Demographic and behavioral characteristics associated with HTLV-1/2 infection among Venezuelan migrants in Central Brazil.

**Variable** ^*^	**Total *n* (%)**	**Positive *n* (%)**	**Negative *n* (%)**	***p***
**Sex**				
Male	122	3 (2.5)	119 (97.5)	0.67
Female	132	2 (1.5)	130 (98.5)	
**Age (years)**				
≤30	159	3 (1.9)	156 (98.1)	1.00
≥31	95	2 (2.1)	93 (97.9)	
**Color**				
White	64	0 (0)	64 (100.0)	0.34
Non White	190	5 (2.6)	185 (97.4)	
Marital status*				
Single/Divorced/Widowed	143	4 (2.8)	139 (97.2)	0.40
Married/Living Together	103	1 (1.0)	102 (99.0)	
Education (years)*				
<10	102	2 (2.0)	100 (98.0)	1.00
≥10	133	3 (2.3)	130 (97.7)	
**Status in Brazil**				
Immigrant	33	0 (0)	33 (100.0)	1.00
Refugee	221	5 (2.3)	216 (97.7)	
**Blood transfusion**				
No or do not know	245	5 (2.0)	240 (98.0)	1.00
Yes	9	0 (0)	9 (100.0)	
Ilicit drug use*				
No	161	5 (3.1)	156 (96.9)	1.00
Yes	8	0 (0.0)	8 (100.0)	
Sexually active*				
No	7	0 (0)	7 (100.0)	1.00
Yes	198	5 (2.5)	193 (97.5)	
Sex for money*				
No	187	5 (2.7)	182 (97.3)	
Yes	1	0 (0)	1 (100.0)	1.00
Use of condoms in the last 12 months*				
No	12	4 (33.3)	8 (66.7)	
Yes	193	1 (0.5)	192 (99.5)	< 0.001
Number of sexual partners in the last 12 months*				
1	190	3 (1.6)	187 (98.4)	
**≥**2	15	2 (13.3)	13 (86.7)	0.044
History of STI*				
No	175	5 (2.9)	170 (97.1)	
Yes	8	0 (0)	8 (100.0)	1.00

*Considered only valid data; STI, sexually transmitted infections.

## Discussion

In this study, most participants were Venezuelans (47.8%) and Haitians (39.7%), reflecting the shift in international migration patterns in the last decade in Brazil (7). After the earthquake in Haiti in January 2010, which caused 230,000 deaths, destroyed buildings, and exacerbated poverty, Brazil became a primary destination for Haitian migrants over the past decade. On humanitarian grounds, Brazil granted Haitian nationals permanent visas and resident permit authorizations (15). Since 2017, there has been an intense flow of Venezuelans to Brazil due to economic and social crises in Venezuela. Most of them initially settled in Roraima (50%) and Amazonas (19%), and through the interiorization program, thousands of Venezuelans have been relocated from Roraima to other Brazilian cities (16). As a result, by 2021, Venezuelans had surpassed Haitians to become the largest group in the formal labor market (7).

This study found a prevalence of anti-HTLV-1/2 antibodies of 0.95% (95% CI: 0.31–2.28) among immigrants and refugees in Central Brazil, HTLV-1 (0.19%) and HTLV-2 (0.76%). Notably, all seropositive individuals were refugees from Venezuela, yielding a prevalence of 2.26% for anti-HTLV-1/2, HTLV-1 (0.45%), and HTLV-2 (1.81%) among Venezuelan refugees. These results align with those reported by Abreu et al. (11), which first revealed the circulation of HTLV-1 and HTLV-2 among Warao indigenous refugees from Venezuela in Belém.

Despite limited data on HTLV-1/2 infection in Venezuela using new screening and confirmatory assays, previous studies detected anti-HTLV-1 and HTLV-2 antibodies among Venezuelans, specifically in blood donors at the Municipal Blood Bank of Caracas (0.11%) (17). Additionally, a prevalence of 0.58% was reported among patients attending the Regional Programmatic Unit of Clinical Immunology from Aragua State (18).

In line with other studies (19–22), which revealed that HTLV-2 infection is endemic in indigenous populations of Venezuela, Abreu et al. (11) also detected a higher frequency of HTLV-2 (2.0%) rather than HTLV-1 (1.0%) among Warao indigenous refugees in Belém. Similarly, the seroprevalence of HTLV-2 (1.81%) among Venezuelan refugees in Central Brazil was higher than that for HTLV-1 (0.45%). It is noteworthy that two individuals in this study were also Warao indigenous Venezuelans. One tested positive for HTLV-1 (M-77) and the other for HTLV-2 (M-69).

Since HTLV-1 and 2 infections are often asymptomatic, these viruses are silently spread from mother to child, mainly by breastfeeding, and through horizontal transmission (1, 4). In fact, all anti-HTLV-1/2 positive individuals reported having been breastfed during childhood, indicating that mother-to-child can be considered one of the modes of transmission among this Venezuelan migrant group. Additionally, all HTLV-1/2 seropositive individuals reported being sexually active, and all but one reported not having used a condom with their sexual partners in the last 12 months, which may contribute to the seropositivity for this infection in the studied population since unprotected sexual intercourse is a significant factor in horizontal HTLV-1/2 infection (23, 24). Indeed, unprotected sexual intercourse in addition to ≥2 sexual partners in the last 12 months were associated with HTLV-1/2 seropositivity among Venezuelans in this study.

Notably, most of the anti-HTLV-1/2 seropositive individuals had a lower educational level. This characteristic was also observed in the study population, which in addition to the cultural and language barriers (most reported difficulties in learning Portuguese, the official language in Brazil; data not shown), may contribute to the high unemployment (35.9%) observed. This cultural and linguistic divide can exacerbate poverty, social discrimination, and marginalization in the group. Consequently, the group’s access to public health services through the free Unified Health System (*Sistema Único de Saúde* - SUS) may be limited.

In this study, although the individuals who were HTLV-1/2 seropositive were clinically asymptomatic at the time of sampling, they were referred for clinical follow-up, as this infection can take many years to develop into severe related diseases. It should be noted that HTLV-1 is associated with ATL and TSP/HAM, as well as other inflammatory diseases (1), and HTLV-2 has been associated with rare cases of myelopathy (2). Therefore, HTLV-1/2 infection is a public health problem, especially in vulnerable populations, such as migrants, and understanding health inequities is essential for implementing effective measures to reduce the burden of disease in this population group (25).

This study has some limitations that should be considered. Initially, the convenience sampling used can compromise the external validity of the results, though the sociodemographic and migration characteristics of the studied population are consistent with those reported elsewhere (26, 27). Also, the challenges of accessing this population and the health restrictions imposed by the COVID-19 pandemic during this study should be noted. These findings are subject to response biases as a limitation of face-to-face interviews. Some strategies were implemented to minimize potential biases, including using previously trained interviewers and private locations for interviews. Whole blood samples were not available to detect HTLV proviral DNA, so anti-HTLV was the only marker used to indicate infection. Therefore, all ELISA reactive samples were confirmed by LIA and typed as HTLV-1 or HTLV-2. Despite these limitations, this study provides valuable epidemiological information, representing the first investigation on HTLV-1/2 infection in immigrants and refugees in Central Brazil, with most of them coming from Latin American countries, especially Venezuela and Haiti. Therefore, more studies on HTLV-1/2 infection among international migrants in Brazil are needed to investigate whether immigration from other endemic countries could increase the rates of HTLV-1/2 associated diseases in Central Brazil, where the expected ATL incidence is low (14 cases per year) (28).

## Conclusion

The findings of this study revealed not only a low seroprevalence of HTLV-1/2 among international migrants in Central Brazil but also the circulation of HTLV-1 and HTLV-2 within the Venezuelan refugee population, an emerging and socially vulnerable group. Therefore, specific health programs should be implemented for immigrants and refugees, mitigating barriers and promoting the implementation of appropriate interventions aimed at the well-being of all international migrants. Considering language and cultural barriers, promoting health, and facilitating early diagnosis of symptoms associated with HTLV-1/2 are some appropriate first steps toward controlling/preventing this infection.

## Data availability statement

The original contributions presented in the study are included in the article/supplementary material, further inquiries can be directed to the corresponding author.

## Ethics statement

The studies involving humans were approved by Committee on Ethics in Research of the Federal University of Goiás. The studies were conducted in accordance with the local legislation and institutional requirements. Written informed consent for participation in this study was provided by the participants' legal guardians/next of kin.

## Author contributions

TAM: Conceptualization, Methodology, Writing – review & editing. LM: Methodology, Writing – review & editing. KS: Methodology, Writing – review & editing. TLM: Methodology, Writing – review & editing. GS: Methodology, Writing – review & editing. ÁS: Methodology, Writing – review & editing. MC: Conceptualization, Methodology, Writing – review & editing. KC: Conceptualization, Methodology, Writing – review & editing. ST: Conceptualization, Formal analysis, Methodology, Writing – original draft. RM: Conceptualization, Supervision, Writing – original draft, Writing – review & editing.
